# Evaluation of Formulation-Dependent Antimicrobial Activity and Plant Compatibility of Chitosan-Based Silver Nanoparticles

**DOI:** 10.3390/md24050183

**Published:** 2026-05-19

**Authors:** Ahmed Hosney, Neringa Matelionienė, Donata Drapanauskaitė, Sana Ullah, Karolina Barčauskaitė

**Affiliations:** Lithuanian Research Centre for Agriculture and Forestry, Instituto Al. 1, LT-58344 Akademija, Lithuania; neringa.matelioniene@lammc.lt (N.M.); donata.drapanauskaite@lammc.lt (D.D.); sana.ullah@lammc.lt (S.U.)

**Keywords:** multifunctional antimicrobial activity, chitosan silver nanoparticles, plant-associated microbes, soil-borne pathogens, spring wheat

## Abstract

Chitosan-based silver nanoparticles (Ch-AgNPs) are emerging as promising antimicrobial materials with potential applications in crop protection. This study evaluated the formulation-dependent antimicrobial activity and plant compatibility of Ch-AgNPs synthesized from chitosan extracted via different routes from shrimp shells. Antibacterial activity was assessed against representative Gram-negative and Gram-positive model bacteria (*Escherichia coli* and *Staphylococcus aureus*), as well as phytopathogenic bacteria (*Xanthomonas campestris*, *Pseudomonas syringae*), using disk diffusion assays. Antifungal activity was evaluated against *Fusarium graminearum* in vitro and in a controlled growth chamber. All formulations exhibited concentration-dependent antibacterial activity, with L10 and L20 formulations derived from optimized lactic acid-based extraction routes and DP4 derived from an inorganic deproteinization-based extraction route showing the highest efficacy at 1.0 mg/mL. Strong antifungal activity was observed, particularly for L10 and DP4, achieving mycelial growth inhibition of 92% and 84%, respectively, at 1.0 mg/mL. Seed germination and seedling growth assays confirmed that all formulations were non-phytotoxic at 1.0 mg/mL, with L10 and DP4 significantly enhancing germination parameters and early plant growth. Under controlled conditions, these formulations also reduced the incidence and severity of crown and root rot in spring wheat caused by *F. graminearum*. These findings demonstrate that optimized Ch-AgNP formulations combine antimicrobial activity with plant compatibility, highlighting their potential for crop protection, pending further environmental safety and agronomic validation under field conditions.

## 1. Introduction

**Figure 1:**
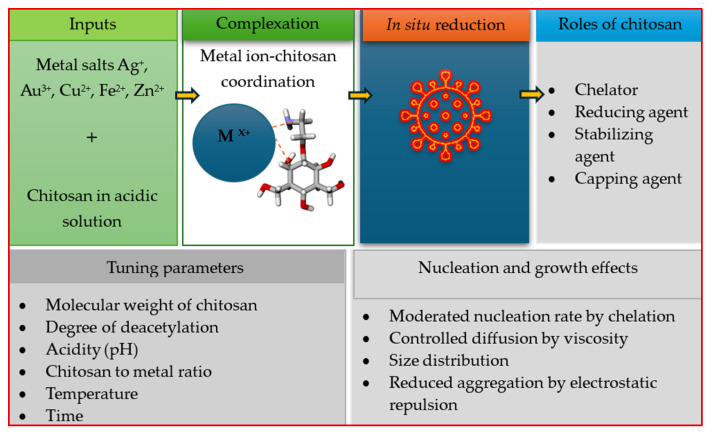
The role of chitosan in green synthesis of metal nanoparticles.

Chitosan metal nanoparticles have emerged as promising green antimicrobial agents for agricultural purposes with wide-ranging antibacterial and antifungal characteristics, biocompatibility, and potential for sustainable applications in crop protection. Chitosan serves as a chelator, reducing and stabilizing agent, as well as a capping agent in nanomaterial synthesis. It has an influence on mechanisms of nucleation, particle size, and dispersion as affected by pH, deacetylation degree, molecular weight, and chitosan to metal ratio [1,2,3,4,5]. These properties enable controlled size distribution and reduced aggregation in green-synthesized nanoparticles (Figure 1). Utilizing chitosan as a green synthesis agent for nanoparticles provides an environmentally friendly approach in which it acts as both a natural stabilizing and reducing agent, thereby eliminating the need for toxic chemicals. Among all chitosan metal nanoparticles, chitosan-based silver nanoparticles (Ch-AgNPs) specifically have attracted the most significant attention, since they combine the intrinsic antimicrobial activity of chitosan and silver nanoparticles, resulting in a synergistic effect [6,7,8,9,10].

Chitosan is a cationic biopolymer that disrupts microbial cell membranes through electrostatic interactions, which lead to leakage of cellular contents and cell death [11,12,13,14,15]. Silver nanoparticles, on the other hand, amplify this effect by generating reactive oxygen species, hindering DNA replication, and liberating Ag^+^ ions that inactivate crucial enzymes and proteins in the pathogens. The small particle size of Ch-AgNPs allows them to penetrate microbial cells, thus enhancing their antimicrobial efficacy [3,16,17,18,19,20,21]. Green synthesis processes for Ch-AgNPs were adopted with plant extracts, fungal beads, or agricultural wastes as reducing and stabilizing agents [22]. These processes produce nanoparticles of controlled size, greater than 44 nm; high crystallinity; and stable colloidal properties [12,23,24,25,26,27,28].

Ch-AgNPs exhibit potent antibacterial effects against a range of phytopathogenic bacteria, including *Pseudomonas syringae*, *Ralstonia solanacearum*, and opportunistic/model bacterium *Serratia marcescens*. Minimum inhibitory concentrations (MICs) as low as 1.5–15 µg/mL have been reported, with enhanced activity compared to either chitosan or silver nanoparticles alone [22,25,29,30,31,32]. In vivo studies demonstrated that Ch-AgNPs reduced disease severity in crops such as tomatoes and stone fruits, maintaining yield, quality, and extending shelf life [12,33]. Ch-AgNPs are effective against major agricultural fungal pathogens, including *Fusarium oxysporum*, *Aspergillus* spp., *Colletotrichum coccodes*, and *Pyricularia* sp. MICs range from 41.7 µg/mL to 1000 µg/mL, with significant inhibition zones observed in vitro [25,28,34,35]. The antifungal mechanism involves disruption of fungal cell walls, chelation of metal ions, and inhibition of enzyme activity [34,35,36].

Ch-AgNPs have been applied as seed coatings, foliar sprays, and antimicrobial films for food packaging and plant protection [34,37,38]. In wheat, chitosan-coated AgNPs have prevented fungal infections during early crop development without phytotoxic effects and even promoted chlorophyll synthesis [25]. In postharvest preservation, Ch-AgNP coatings on tomatoes and litchis have reduced microbial spoilage and maintained fruit quality, with silver migration remaining within safety limits [12,39]. Nanocomposite films incorporating Ch-AgNPs offer controlled release, prolonged antimicrobial action, and low cytotoxicity, making them suitable for food packaging [12,40,41]. The combination of chitosan and silver nanoparticles results in enhanced antimicrobial activity and reduced cytotoxicity compared to bare AgNPs. Chitosan capping stabilizes AgNPs, minimizes aggregation, and improves biocompatibility, as evidenced by low toxicity to plant tissues and mammalian cells [42,43,44]. Synergistic effects are also observed when Ch-AgNPs are combined with essential oils or antibiotics, further broadening their antimicrobial spectrum [42,43].

Despite the widespread studies on the antimicrobial activity of chitosan–silver nanoparticles, there is a lack of assessment of how the chitosan extraction methods and the formulation of chitosan-based silver nanoparticles influence antimicrobial efficacy. These studies have primarily focused on in vitro antimicrobial activity testing using a single formulation of Ch-AgNPs derived from a single chitosan extraction method, with limited attention to formulation-dependent performance, plant compatibility, and translation into realistic plant pathogen systems. For instance, the correlation between in vitro antifungal efficacy and in vivo suppression of soil-borne diseases, such as crown and root rot of wheat caused by *Fusarium graminearum*, remains inadequately investigated, primarily due to the limited evaluation of seed germination and early seedling responses to antifungal treatments. Additionally, most studies do not integrate antibacterial, antifungal, and plant response assessments within a single experimental framework. To the best of our knowledge, this research was performed to provide a comprehensive assessment linking chitosan extraction routes, nanoparticle formulation, and multifunctional antimicrobial performance, including antibacterial, antifungal, plant compatibility, and in vivo disease suppression against *Fusarium graminearum* in spring wheat.

Therefore, the present research provides an integrated evaluation of formulation-dependent antimicrobial activity and plant compatibility to assess the influence of chitosan extraction methods and nanoparticle formulations on antimicrobial efficacy against bacterial and fungal pathogens in vitro, as well as validating their disease suppressive potential in a pot experiment, evaluating the incidence and severity of crown and root rot in spring wheat.

## 2. Results

### 2.1. Antibacterial Activity Evaluation

Chitosan–silver nanoparticle formulations at concentrations of 0.2, 0.5, and 1.0 mg/mL were evaluated for antibacterial efficacy against the Gram-negative bacteria *Escherichia coli*, *Xanthomonas campestris*, and *Pseudomonas syringae* (Table 1) and Gram-positive *Staphylococcus aureus* (Table 2) using the disk diffusion method. Antibacterial activity was observed in all formulations, with increasing concentration generally leading to increased inhibition and statistically significant differences among formulations and concentrations, as determined by Tukey’s HSD (*p* < 0.05). On the other hand, the positive control tetracycline at 0.5 μg/mL produced inhibition zones of 22 mm for *E. coli*, 46 mm for *X. campestris*, 37 mm for *P. syringae*, and 26 mm for *Staphylococcus aureus*, indicating the maximum antibacterial activity possible under agar diffusion conditions.

Antibacterial efficacy of chitosan–silver nanoparticles formulations showed that L20 formulation exhibited high inhibition against *E. coli* at low to moderate concentrations (0.2–0.5 mg/mL), yielding significantly higher inhibition zones than the other formulations, while L10 and L20 reached maximum activity (≈13–14 mm) at 1.0 mg/mL. On the other hand, LH1 and AS3 had a consistently weak inhibition. In the case of *X. campestris*, the differences were formulation-related, with almost no inhibitory effect at a concentration of 0.2 mg/mL. However, L10 was the most efficient formulation at 0.5 mg/mL concentration, resulting in the largest inhibition zone (up to 19.37 mm) at 1.0 mg/mL, and was greatly favored over the other formulations. Clear responses were observed for *P. syringae*, with DP4 and AS3 resulting in large inhibition zones (≈14.5–21 mm) at 0.5–1.0 mg/mL. Therefore, it is obvious that antibacterial efficacy is influenced not only by the concentration of the nanoparticles but also by the formulation, which is based on the chitosan extraction method.

In contrast, the antibacterial efficacy of chitosan–silver nanoparticle formulations against Gram-positive *Staphylococcus aureus* showed lower inhibition zones than those in Gram-negative bacteria, as depicted in Table 2. The lowest concentration (0.2 mg/mL) of all formulations resulted in almost identical small inhibition zones (≈6.0–6.5 mm) without any significant statistical differences, thus confirming that the dose was insufficient to induce strong antibacterial action. The result of increasing the concentration to 0.5 mg/mL showed a formulation-related response, with a significantly larger inhibition zone (9.0 ± 0.0 mm) in DP4 than in other formulations, reflecting improvements in silver bioavailability or diffusion properties of DP4. At 1.0 mg/mL, DP4 exhibited the highest inhibition (13.0 ± 0.81 mm), compared to L10 (12.63 ± 1.25 mm) and L20 (10.0 ± 2.8 mm), while LH1 and AS3 remained significantly less effective (≈8.75 mm).

Recent research has reported that chitosan-stabilized silver nanoparticles may exhibit enhanced antibacterial activity against Gram-negative bacteria, attributed to improved nanoparticle stability, Ag^+^ release, and electrostatic interactions with the negatively charged bacterial membranes. In contrast, the absence of the outer membrane in Gram-positive *Staphylococcus aureus* and the presence of a thick cell wall hinder nanoparticles’ penetration into the bacterial cell [36,43,44,45]. However, it should be noted that inhibition zone measurements obtained in the present research reflect both the antimicrobial activity and diffusion characteristics of the tested formulations; therefore, direct comparisons of susceptibility between Gram-positive and Gram-negative bacteria should be interpreted with caution and within the context of the specific tested formulations, rather than relying solely on inhibition zone measurements.

However, all formulations exhibited lower absolute zones of inhibition than those of tetracycline against bacterial species (*E. coli*, *X. campestris*, and *P. syringae*). This reflects the major methodological and mechanistic differences between small-molecule antibiotics and chitosan–silver nanoparticle-based antimicrobials. Tetracycline can rapidly diffuse through the agar and inhibit protein synthesis. While the chitosan–silver nanoparticles diffuse with a slower rate of silver ion release, they cause membrane disruption and induce oxidative stress. A similar observation has been reported in recent studies, in which chitosan–silver nanocomposites tested at similar mg/mL concentrations exhibited smaller agar diffusion zones than antibiotics, despite exhibiting potent bactericidal efficacy in broth microdilution and time-kill assays [22,46]. Therefore, the smaller zones of inhibition observed consistently in this research, as compared to tetracycline, did not relate to any biologically relevant effect but instead to the contrast between the different modes of action and diffusion behaviors.

### 2.2. Antifungal Activity Evaluation

#### 2.2.1. Antifungal Activity In Vitro

The antifungal efficacy of all chitosan–silver-based nanoparticle formulations was directly proportional to concentration against *Fusarium graminearum*, as represented by reduced mycelial diameter and increased growth inhibition (Table 3). The antifungal activity of all chitosan–silver nanoparticle formulations at a concentration of 0.2 mg/mL was absent, with no growth inhibition, confirming that this concentration fell below the effective limit needed to inhibit *Fusarium graminearum* fungal growth. In contrast, at a dose of 0.5 mg/mL, the formulations L10, DP4, and CC were able to reduce mycelial diameter significantly to (48 ± 14.09 mm), (73 ± 6.16 mm), (66 ± 18.49 mm), while attaining 40%, 8.75%, and 17.50% growth inhibition, respectively. On the other hand, the formulations LH1, L20, and AS3 remained inactive. The highest antifungal efficacy of chitosan–silver nanoparticle formulations was recorded at 1.0 mg/mL, with L10 demonstrating the strongest efficacy (mycelial diameter 6.50 ± 13.0 mm; 92% inhibition) near complete inhibition, followed by DP4 (12.5 ± 17.45 mm; 84%) and L20 (14.75 ± 18.1 mm; 81.56%), while CC and AS3 showed a moderate inhibitory effect (60% and 52.5%, respectively), and LH1 exhibited the weakest effect against *Fusarium graminearum*. The antifungal activity was assessed in comparison with metconazole, a commonly used fungicide that exhibited complete inhibition (100%). Although the Ch-AgNP formulations did not achieve equivalent inhibition, they demonstrated substantial suppression of *Fusarium graminearum*, particularly at 1.0 mg/mL, indicating their effectiveness as promising antifungal candidates under controlled conditions. Relatively high standard deviation values were observed in the treatments (L10, L20, DP4, and AS3) at 1.0 mg/mL due to variability among four replicates, where complete inhibition (0 mm) occurred in most cases, while occasional partial fungal growth was recorded in some individual replicates. This reflects biological variability rather than experimental error.

The observed reduction in mycelial growth at higher Ch-AgNPs concentrations is consistent with previous reports indicating that chitosan–silver nanoparticle systems inhibit fungal development through the combined effects of membrane disruption, oxidative stress induction, and controlled Ag^+^ release [22,45,46,47,48,49]. Moreover, we observed the important role of formulation design in achieving concentration-dependent effects and their efficacy through overcoming the cell wall structural complexities of *Fusarium graminearum*. Furthermore, some studies have reported that silver nanoparticles combined with chitosan matrices deliver better antifungal performance than silver nanoparticles used in their unbound state because the chitosan matrix enhances both nanoparticle stability and Ag^+^ ion release control and enhances contact with fungal cell wall and membrane structures. The effects arise from the cell surface proteins, glucans, and membrane structures rather than direct electrostatic interaction with chitin [22,46,49].

Other studies have shown that silver nanoparticles can disrupt fungal cell membrane integrity, leading to increased permeability, mitochondrial dysfunction, and the generation of reactive oxygen species (ROS), which ultimately inhibit fungal growth. In addition, chitosan contributes to antifungal activity by interacting with cell wall components, altering membrane permeability, and inhibiting spore germination [36,39,48]. The highest significant effectiveness of L10 and DP4 at a concentration of 1.0 mg/mL suggests that these formulations have a more efficient balance between silver bioavailability and diffusion within the chitosan matrix, consistent with data indicating that optimized chitosan–silver nanocomposites can achieve greater than 80% inhibition of *Fusarium* spp. mycelium in vitro [2,13,19,21,25,27,36,47,49,50,51,52,53]. Thus, these results reaffirm that chitosan–silver nanoparticle formulations (particularly L10 and DP4) could be promising antifungal agents against *F. graminearum* and might be used in sustainable wheat crop protection systems as alternative fungicides in the future after environmental safety and agronomic validation.

#### 2.2.2. Seed Germination Test

**Figure 2:**
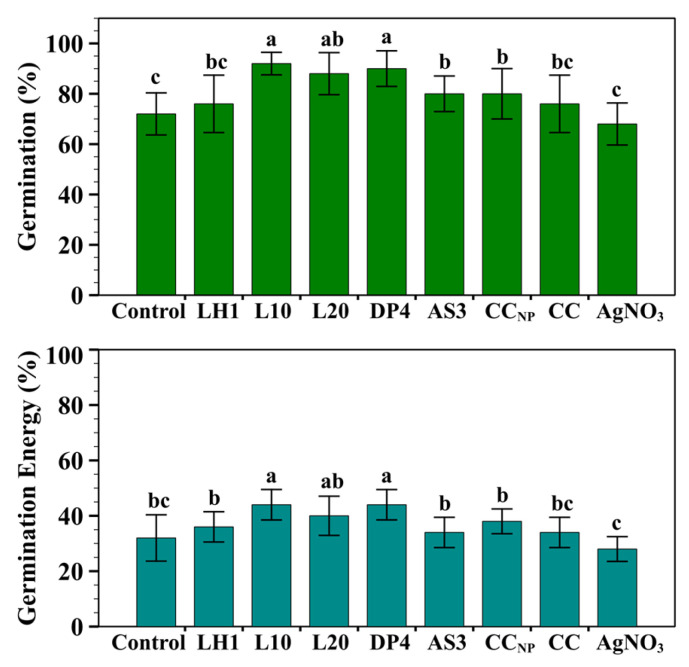
Germination percentage of wheat seedlings at 1.0 mg/mL. Values are expressed as mean ± standard deviation of five replicates (*n* = 5). Different letters indicate significant differences among treatments as determined by a one-way ANOVA followed by Tukey’s HSD test (*p* < 0.05).

A seed germination test was conducted on wheat seeds before post-emergence tests for antifungal activity against *Fusarium graminearum* under controlled growth chamber conditions. All formulations of chitosan–silver nanoparticles, commercial chitosan, and silver nitrate salt were applied to wheat seeds at a concentration of 1.0 mg/mL, which was the most effective concentration obtained from an in vitro antifungal evaluation experiment. Seed germination and germination energy percentages were assessed to ensure plant compatibility (Figure 2) and identify any harmful effects on germination. At this concentration, no formula caused any phytotoxic effects, but significant formulation-dependent improvements in germination parameters were observed relative to the untreated control.

As shown in Figure 2 the formulations L10 and DP4 exhibited the maximal values of germination percentage (92 ± 4.47% and 90 ± 7.07%, respectively) and germination energy percentage (44 ± 5.50% and 44 ± 5.48%, respectively), reflecting enhanced seed viability and quick early rooting. These was followed by moderate stimulation in the L20 formulation (88 ± 8.36 GP%, 40 ± 7.07 GE%), whereas AS3, CC_NP_, LH1 and CC were lower than other formulations and moderately higher than control (72 ± 8.36% germination; 32 ± 8.36% energy). Silver nitrate salt (AgNO_3_) exhibited the lowest GP% and GE%, indicating a less favorable effect compared with commercial chitosan and chitosan-based silver nanoparticle formulations. These results suggest that chitosan–silver nanoparticle formulations at a concentration of 1.0 mg/mL improved the physiological processes associated with early germination, with the most effectively enhanced germination capacity and early seedling vigor using L10 and DP4.

Similar stimulatory effects on germination and viability of cereal seeds have been widely discussed in recent research, attributed to increased antioxidant capacity induced with chitosan and chitosan–silver nanoparticles, usually in combination with non-toxic concentrations of Ag^+^ ions [2,19,25,36,51,52,53]. Notably, previous studies suggested that excessive concentration or poorly stabilized silver nanoparticles can inhibit seed germination, leading to oxidative stress and adverse consequences [36,39,46]. Hence, increased germination and energy percentages in the present study indicated the safety and suitability of physio-chemical conditions for the fungicidal evaluation using a 1.0 mg/mL chitosan–silver nanoparticle treatment against *F. graminearum* pathogen in a controlled chamber environment.

#### 2.2.3. Shoot and Root Lengths

**Figure 3:**
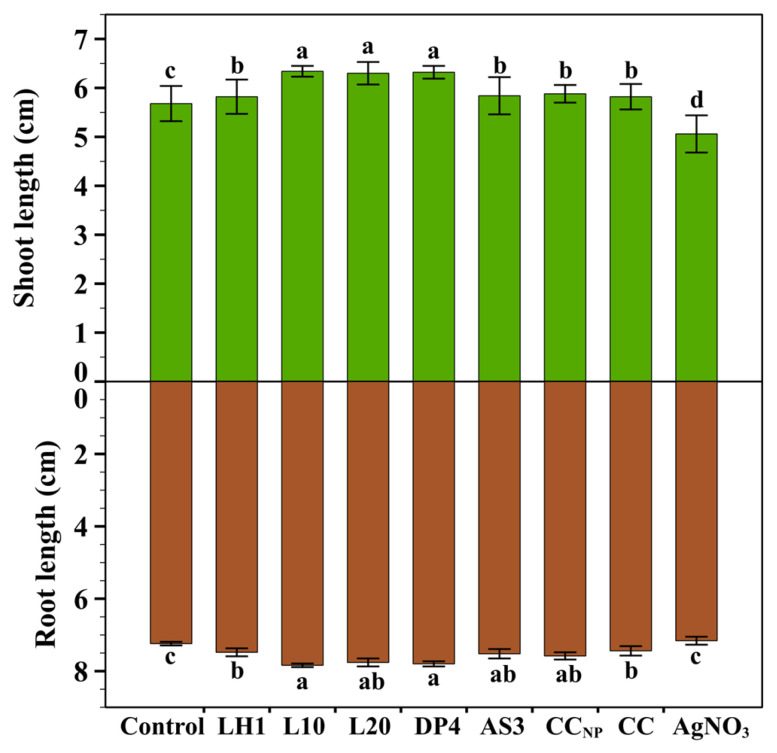
Shoot and root lengths (cm) of wheat seedlings at 1.0 mg/mL. Values are expressed as mean ± standard deviation of five replicates (*n* = 5). Different letters indicate significant differences among treatments as determined by one-way ANOVA followed by Tukey’s HSD test (*p* < 0.05).

Shoot and root lengths of the seedlings treated with 1.0 mg/mL of each chitosan–silver nanoparticle formulation were evaluated (Figure 3). Root and shoot lengths of treated seeds were almost significantly different compared to those of the untreated control (Figure 3). L10 and DP4 formulations significantly enhanced shoot length (6.34 ± 0.11 cm and 6.32 ± 0.13 cm, respectively) and root length (7.84 ± 0.05 cm and 7.80 ± 0.07 cm, respectively), thus showing they had a significant effect in promoting early seedling growth. Moderate improvement in the shoot and root lengths was shown by formulation L20 (6.30 ± 0.23 cm, 7.76 ± 0.11 cm), respectively. On the other hand, the formulations CC_NP_, LH1, CC, and AS3 showed a significant moderate effect in promoting growth compared to the control. Whereas silver nitrate (AgNO_3_) demonstrated the shortest shoot and root lengths, indicating a suppressive effect on the roots and shoots of spring wheat compared with commercial chitosan and chitosan-based silver nanoparticle formulations.

These results indicate that almost all chitosan–silver nanoparticle formulations at a concentration of 1.0 mg/mL were not phytotoxic to the seedlings but rather promoted plant growth, particularly root elongation, which is vital for nutrient and water absorption, even if L10 and DP4 had the best performance when achieving a fine balancing act between silver functionality and plant physiological tolerance. The results of the current research agree with previous studies, which revealed that chitosan–silver nanoparticles and nanocomposites can stimulate root architecture and shoot biomass without being destructive through the generation of sufficient oxidative stress [2,19,51,52].

#### 2.2.4. Antifungal Activity of Ch-AgNPs Against *F. graminearum* in a Controlled Chamber

**Figure 4:**
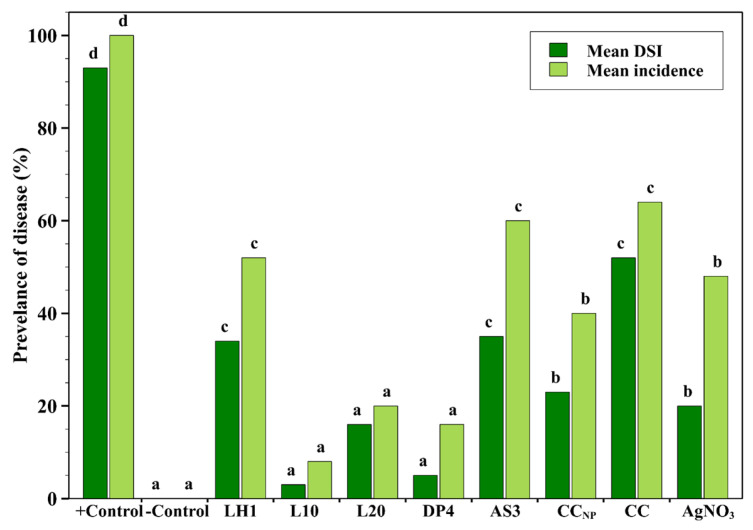
Incidence and disease severity indices of *Fusarium graminearum* in spring wheat under Ch-AgNP formulations at 1.0 mg/mL. Grouped bars represent the mean disease severity index (DSI) and disease incidence. Different letters above bars indicate statistically significant differences according to Tukey’s HSD test (*p* < 0.05).

The effects of commercial chitosan and chitosan–silver nanoparticle formulations at 1.0 mg/mL on crown and root rot of spring wheat caused by *Fusarium graminearum* were evaluated under controlled conditions in a growth chamber (Figure 4). The highest incidence (100%) and severity indices (93%) of crown and root rot disease were recorded in the positive control group. In contrast, the negative control group exhibited no symptoms, thereby validating the efficiency of this chamber’s experimental system.

On the other hand, DP4 and L10 formulations exhibited the lowest crown and root rot incidence and severity indices, with the lowest mean DSI attributed to treatments with statistically low disease severity indices. Subsequently, the L20 formulation significantly inhibited disease development, but with lower efficacy compared to L10 and DP4. The CC_NP_ AgNO_3_ also showed moderate inhibition of crown and root rot. Conversely, the CC, AS3, and LH1 formulations demonstrated high disease occurrence, suggesting poor control of crown and root rot caused by *Fusarium graminearum*. However, each group of commercial chitosan and silver nitrate (AgNO_3_) separately showed the least efficacy compared with chitosan–silver nanoparticle formulations, implying the value of their combination stability and transport for disease management [42,43].

The results of the present research clearly show that chitosan–silver nanoparticle formulations, especially DP4 and L10, significantly suppressed the incidence of crown and root rot on spring wheat under controlled chamber conditions. This is consistent with recent studies, which reported that chitosan-based nanocomposites can adhere to fungal hyphae, disrupt cell wall integrity, and enhance the controlled release of Ag^+^ ions, leading to membrane damage, mitochondrial oxidative dysfunction, and fungal dysfunction [19,39,42]. Evidence for the inhibitory effects of DP4 and L10 against crown and root rot strengthens existing knowledge of the antifungal mechanisms of chitosan-based silver nanoparticles and of in vivo *studies* of *Fusarium infections* in plants. These observations confirm that optimized formulations of chitosan and silver nanoparticles have successfully controlled crown and root rot of spring wheat. They thus present themselves as potential candidates for an integrated control strategy against soil-borne *Fusarium graminearum*; however, a comprehensive environmental assessment is still needed, including the evaluation of silver residue accumulation in plant tissues, impacts on soil microbial communities, and the environmental fate of Ch-AgNPs under field conditions. The selection of a single concentration (1.0 mg/mL) for the seed and pot experiments was based on its in vitro efficacy; however, it represents a limitation of this study. Further research is needed to incorporate a range of concentrations to create dose–response relationships to identify the optimal application rates under field conditions.

It should be noted that the pot experiment was conducted under controlled growth-chamber conditions, which do not fully demonstrate the environmental variability encountered in field conditions. Factors such as light exposure, humidity, soil pH, and organic matter content may significantly influence the stability of chitosan–silver nanoparticles, the release of silver ions, and their antimicrobial efficacy. Therefore, the results obtained in this study should be interpreted as a proof of concept under controlled conditions, and further validation under field conditions is needed.

## 3. Discussion

The present research demonstrates that the antimicrobial ability and plant protective capacity of Ch-AgNPs are influenced by the chitosan extraction method used to prepare each Ch-AgNPs formulation. The differences in antimicrobial efficacy among Ch-AgNPs formulations are likely related to extraction-dependent variations in chitosan structure and nanoparticle formation behavior. Although the molecular weight and polydispersity index were not determined in the present study, the extraction conditions of chitosan may have influenced polymer chain structural organization, residual mineral content, availability of amino and hydroxyl functional groups, and chitosan–silver interactions during the nanoparticle synthesis phase. These factors can affect the nucleation and stabilization of silver nanoparticles, resulting in differences in bioactivity. In the present study, Ch-AgNPs-formulations DP4 (55 nm) and L10 (64 nm) produced more biologically effective nanoparticles, which may explain their enhanced antimicrobial activity. Smaller nanoparticles generally possess a higher surface area, improved interaction with microbial cells, and greater Ag^+^ release efficiency, thereby accelerating biological activity. Nevertheless, comprehensive structural characterization including molecular weight, polydispersity index, zeta potential, silver loading, and release kinetics is still required in future research to fully establish the structure–activity relationships of these chitosan–silver nanoparticle formulations. Although the extracted chitosan samples exhibited high degrees of deacetylation (>95%), the different formulations showed distinct biological responses, suggesting that the DD parameter alone cannot explain the observed antimicrobial activity [12,23,24,25]. Instead, the observed antimicrobial differences are more likely related to the combination of extraction conditions, such as acid type and concentration during the demineralization step, NaOH treatment during deproteinization and deacetylation phases, reaction temperatures, prolonged alkaline soaking time, residual ash levels, and particle size, which led to chitosan–silver interactions [26,27,28]. Therefore, the different formulation-dependent antimicrobial activities observed in this study should be interpreted in terms of multiple physicochemical factors rather than a single structural characteristic.

The organic demineralization extraction route using lactic acid produced markedly different biological responses because of varying acid concentrations and temperatures. For instance, the LH1 sample (1.0 mg/mL) prepared from 1% lactic acid at room temperature exhibited weak antimicrobial activity. This may be related to mild acid concentration during demineralization, which may have resulted in incomplete mineral removal and limited the availability of functional groups for silver binding and nanoparticle stabilization. In contrast, L10 (1.0 mg/mL) extracted using 10% lactic acid at 50 °C exhibited a significantly efficient antimicrobial activity, particularly effective against *X. campestris*, *F. graminearum*, and spring wheat crown and root rot. This suggests that a moderate organic acid concentration combined with elevated temperature enhanced shell matrix disruption and demineralization efficiency while maintaining chitosan reactivity without damaging the polymer structure [29,30]. The extended 24 h soaking period during the deproteinization and deacetylation stages may have enhanced the removal of protein and acetyl groups and led to better interaction between chitosan and silver ions. L20 showed significant antibacterial activity against *E. coli* and antifungal activity at 1.0 mg/mL, but said activity was still lower than that of L10 throughout testing. This may indicate that excessive acidic demineralization can change the chitosan chain structure, which affects nanoparticle dispersion and diffusion behavior [26,29,31,32].

The AS3 (1.0 mg/mL) formulation, which was extracted using 3% acetic acid, demonstrated selective antibacterial activity against *P. syringae* but showed reduced efficacy against *E. coli*, *S. aureus*, and *F. graminearum*. However, acetic acid commonly functions as a chitosan dissolving agent; its role during extraction may result in chitosan with different characteristics compared with lactic acid extraction methods [29,33]. The moderate activity of AS3 against *P. syringae* demonstrates that the chitosan matrix from extraction might have produced stabilized silver nanoparticles but failed to deliver effective antimicrobial protection against a broad spectrum of tested species. On the other hand, DP4 (1.0 mg/mL), which was extracted using an inorganic method, starting with NaOH deproteinization followed by HCl demineralization and NaOH deacetylation, showed significant antimicrobial activity against *P. syringae*, *S. aureus*, and *F. graminearum* and was one of the most effective formulations during the pot experiment. The high antimicrobial performance of DP4 might be related to the efficient mineral removal by the HCl during demineralization, resulting in lower ash content, smaller nanoparticle size, and increased silver availability [24,26]. DP4 possessed a smaller particle size (55 nm) than L20 (66 nm), which may have increased surface area and enhanced silver ion release and interaction with microbial cell surfaces [24].

The antimicrobial properties of Ch-AgNP formulations at (1.0 mg/mL) may be explained by the combined effects of chitosan and silver interaction [11,12,16,23]. Chitosan, as a biopolymer, can interact with microbial surfaces through its positively charged amino groups. The AgNPs and released Ag^+^ ions can disrupt membrane integrity, which leads to cellular metabolic interference and oxidative stress induction [13,34,35]. Each Ch-AgNPs formulation in bacterial assays showed different antibacterial activity against each bacterial strain. L20 and L10 achieved maximum effectiveness against *E. coli*, while L10 was efficient against *X. campestris*, and DP4 and AS3 performed efficiently against *P. syringae*, while DP4 and L10 had a moderately significant effect against *S. aureus*. The study results demonstrate that antimicrobial activity depends on both nanoparticle concentration and the formulation-specific interactions that occur between chitosan, silver, and microbial cell structures in each formulation. However, it should be noted that the interpretation of results for comparing Gram-negative and Gram-positive susceptibility should be cautious, because the disk diffusion reflects both antimicrobial effectiveness and diffusion through agar [3,36,37,38,39,40].

Moreover, the antifungal activity evaluation supported the importance of the Ch-AgNPs’ formulation design. All formulations at a 0.2 mg/mL concentration had no significant effect to suppress *F. graminearum* growth, reflecting that this concentration has fallen below the effective threshold. In contrast, the formulations L10, DP4, and L20 at a 1.0 mg/mL level showed significantly strong mycelial growth inhibition, while LH1, AS3, and CC exhibited weak performance. The findings suggest that antifungal agents need both silver bioavailability and a specific chitosan matrix to achieve their full effectiveness [22,36]. Fungal cell walls exhibit complex structures that combine chitin with glucans, proteins, and other polymers, which limit the penetration of nanoparticles into their structure [18,22,41]. Therefore, formulations that exhibit better dispersion and smaller particle sizes with superior Ag^+^ release capabilities are likely to achieve more significant antifungal effects [12,21,36,38].

Furthermore, the research showed that Ch-AgNP formulations reached their optimal level at 1.0 mg/mL, which demonstrated non-toxic effects on spring wheat seed germination and seedling development. The L10 and DP4 treatments increased all germination attributes by more than AgNO_3_, which showed lower effectiveness than the untreated control group. Chitosan-based stabilization decreased ionic silver phytotoxicity while it preserved its antimicrobial properties [2,42,43]. Chitosan enhances plant compatibility through its ability to boost water absorption, which triggers early plant development and strengthens root growth [4,5,44,45]. The superior plant response observed for L10 and DP4 reflects a significant balance between silver functionality and chitosan biocompatibility. Recent studies demonstrate that silver nanoparticles can either stimulate or inhibit plant growth depending on five factors, which are particle size, dose, coating material, exposure duration, and plant species [18,19,28,47,48]. It is emphasized that AgNPs may show potential to boost seed germination and photosynthetic efficiency and initial plant growth during the proper concentration range, but their excessive exposure results in phytotoxic effects [18,19,28,47,48]. Other studies found that AgNO_3_ treatment led to weaker seedling growth than Ch-AgNP formulations, which showed that free silver ions induced more severe stress on wheat seedlings than silver in chitosan matrix form [2,21,38,46,49]. This agrees with the concept that polymer-stabilized nanoparticles can control metal ion release while reducing sudden toxicity and enhancing their biological compatibility [11,23].

The pot experiment in a controlled growth chamber confirmed a reduction in crown and root rot caused by *F. graminearum*, further indicating that the most effective Ch-AgNP formulations retained biological activity beyond agar-based in vitro assays. In particular, the L10 and DP4 Ch-AgNP formulations maintained their antifungal properties in a plant system and successfully reduced disease progression during controlled testing. This is relevant because crown and root rot diseases caused by *Fusarium* species occur at the soil–root and crown interface, where antimicrobial agents must remain active in the presence of plant tissues, substrate components, moisture variation, and microbial competition [2,18,21,38]. Recent studies demonstrate that chitosan and its nanomaterial derivatives can be effective against *Fusarium* diseases because they possess direct antifungal capabilities and boost the host’s protective responses [38,50]. It has been reported that chitosan hydrochloride effectively inhibited *F. graminearum* development by decreasing the expression of genes involved in fungal growth, respiration, and virulence, as well as the trichothecene biosynthetic pathway [19,21,38]. These findings establish that chitosan-based systems, together with other nano-enabled methods, represent effective solutions for controlling fungal diseases that affect soil and cereal crops, while serving as sustainable methods for controlling harmful *Fusarium* fungi.

## 4. Materials and Methods

Chitosan–silver nanoparticles (Ch-AgNPs) used in this research contribution were obtained from chitosan samples isolated from the shells of white leg shrimps *(Litopenaeus vannamei*) purchased from the local market (Kaunas, Lithuania). The shells were thoroughly washed, dried, and ground before isolation via different extraction routes [45,54]. Commercial chitosan purchased from Santa Cruz Biotechnology (Dallas, TX, USA) had the following specifications: ash content > 1%, moisture content < 0.5%, and degree of deacetylation > 91% [45]. The categorization of chitosan extracted from shrimp shells and the corresponding chitosan–silver-based nanoparticles prepared via microwave-assisted method is described in Table 4. The samples were obtained via different extraction conditions, resulting in variations in physicochemical properties. The extracted chitosan samples exhibited low moisture (0.19–2.74%) and ash content (0.70–3.50%), with a high degree of deacetylation (>95%) across all samples, exceeding that of commercial chitosan (>91%), which could be explained by prolonged exposure to NaOH during deproteinization and deacetylation stages, respectively. It should be noted that the degree of deacetylation (DD) of extracted chitosan samples was determined using the FTIR spectroscopic method based on the absorbance ratio of characteristic amide and hydroxyl bands (~1655/3450 cm^−1^); therefore, the reported (DD) values should be interpreted as indicative of highly deacetylated chitosan rather than exact quantitative estimation. The molecular weight of chitosan and the loading of silver were not determined in the present research; however, identical synthesis conditions were applied to all formulations to ensure comparability. Chitosan–silver nanoparticles were synthesized by mixing 10 mM silver nitrate with 1% (*w*/*v*) chitosan solution (prepared in 2% acetic acid), followed by the addition of 10% (*w*/*v*) ascorbic acid as a reducing agent, then the pH was adjusted to 5.5 using a diluted NaOH solution under continuous stirring before microwave treatment. The reaction was conducted under microwave irradiation (600-Watt, 5 min), and the resulting nanoparticles were collected by centrifugation, washed, and dried. Further details on extraction procedures and physicochemical characterization are provided in our previous research [45,54], which confirmed nanoparticle formation and structural variation among samples. The particle size distribution of prepared samples ranged from approximately 55 nm (DP4) to 66 nm (L20); intermediate sizes were observed for L10 (64 nm), AS3, and LH1 (62 nm). UV–Vis spectroscopy revealed characteristic surface plasmon resonance peaks at 420–440 nm, while FTIR and RAMAN analyses indicated interactions between chitosan functional groups and silver ions with noticeable shifts in peak positions after nanoparticle formation. XRD patterns confirmed the crystalline structure of metallic silver with characteristic diffraction planes (111), (200), and (220) (JCPDS 04-0783) [55].

PROFI 2: A professional peat-based substrate was purchased from the local market (Kaunas, Lithuania) and consisted of 60% medium-fraction light Sphagnum peat (0–20 mm) and 40% dark humus peat (0–5 mm). The substrate was enriched with limestone, NPK fertilizers, micronutrients, a water absorption regulator, clay to enhance nutrient retention, and root-stimulating additives, providing balanced nutrient availability for up to six weeks. The material had a pH of 5.5–6.0, electrical conductivity of 1.1–1.6 mS/cm, moisture content of 45–55%, and nutrient concentrations of 150–230 mg/L N, 170–260 mg/L P, and 190–300 mg/L K. Furthermore, spring wheat seeds of the Zenon variety were tested in the pot experiments.

### 4.1. In Vitro Antibacterial Assay

Four bacterial strains were selected for the current research, which involved *Escherichia coli* and *Staphylococcus aureus* from the Microbiology Laboratory collection of the Lithuanian Research Centre for Agriculture and Forestry, and *Xanthomonas campestris pv. campestris* (LMG 567) and *Pseudomonas syringae* (LMG 1247) obtained from the BCCM/LMG collection. *Escherichia coli and Staphylococcus aureus* were selected to be used in the antibacterial activity assay as model bacteria because they serve as standardized, rapid-growing model organisms to evaluate the broad-spectrum antibacterial activity of Ch-AgNP formulations. Each strain was maintained on nutrient agar slants at 4 °C, revived just before testing by culturing them on Plate Count Agar (PCA) media at 37 °C for 24 h.

The antibacterial activity of chitosan-based silver nanoparticles (Ch-AgNPs) was assessed by applying the agar disk diffusion method against bacterial isolates. Plate count agar (PCA) was used for all bacterial susceptibility assays. Bacterial inocula were prepared from 24-hour-old cultures, and the turbidity was adjusted to 0.5 McFarland standard (≈1 × 10^8^ CFU mL^−1^). Two milliliters of the bacterial suspension were added to 200 mL of molten PCA cooled to 40 °C, mixed thoroughly, and 10 mL aliquots were poured into Petri dishes. After solidification, six-millimeter sterilized disks were placed on the surface of each inoculated agar plate, ensuring adequate space between disks so that their inhibition zones did not overlap. The dried Ch-AgNP samples were dispersed in sterile distilled water to prepare the required concentrations. Subsequently, each disk received 20 µL of chitosan-based silver nanoparticle suspension (0.2, 0.5, and 1 mg mL^−1^) applied directly onto the disk surface. For positive control, 20 µL tetracycline (0.5 µg mL^−1^) was used, while sterile double-distilled water (20 µL) was applied as a negative control to the blank disk. All tests were in four replicates for each treatment, and the bacteria involved in the study allow reproducibility.

The disk application was followed by inoculating the petri plates with *E. coli* and *S. aureus*, then incubating at 37 °C for 24 h. Inoculation with plant-pathogenic bacteria was carried out with *P. syringae* and *X. campestris* and then incubated at 28 °C for 48 h, owing to optimal growth temperature and slow growth rates. After the incubation period, the antibacterial efficacy was assessed by measuring clear inhibition zone diameters surrounding each disk, including the diameter of the paper disk. Inhibition zones were measured to the nearest millimeter using a ruler. A distinct circular clear zone indicated inhibition of bacterial growth by the Ch-AgNP formulations or antibiotic control, while the absence of an inhibition zone around the negative control disk containing sterile distilled water confirmed that the solvent itself had no antibacterial activity.

### 4.2. In Vitro Antifungal Assay

One fungal pathogen isolate, *Fusarium graminearum* 13121 (Microbiology laboratory collection, Lithuanian Research Centre for Agriculture and Forestry), was used for antifungal assay. The *Fusarium graminearum* fungus was cultivated on fresh potato dextrose agar (PDA) media and incubated at 25 °C for 6 days to obtain actively growing mycelial colonies. PDA medium (Liofilchem, Roseto degli Abruzzi, Italy) was prepared according to the manufacturer’s instructions (42 g powder in 1 L deionized water) and sterilized by autoclaving at 121 °C for 15 min. After this process, molten PDA was cooled down to approximately 45 °C before mixing with nanoparticles and fungicide treatments. Stock suspensions of Ch-AgNPs (10 mg/mL) were prepared in sterile distilled water, and appropriate volumes (0.2, 0.5, and 1.0 mL) of each suspension were incorporated into molten PDA medium to obtain final concentrations of 0.2, 0.5, and 1.0 mg/mL. The suspensions were mixed with 10 mL of PDA medium per plate at approximately 45 °C before solidification to ensure homogeneous nanoparticle distribution throughout the medium.

Subsequently, Ch-AgNPs were tested for antifungal activity against *F. graminearum* using a mycelial growth inhibition assay on PDA. Metconazole (25 μg/L) was used as a positive control, and sterile double-distilled water (20 µL) served as a negative control. After agar solidification, a 5 mm mycelial plug was cut from the margin of a 6-day-old *F. graminearum* culture in PDA and centrally inoculated onto each plate. The plug was placed with mycelium at the center of the plate to promote direct contact with the treated medium. The inoculated plates were incubated at 25 °C for 6 days, and the diameters of mycelial growth and the percentages of growth inhibition were calculated. All treatments were prepared with four replicates for each treatment and fungus.

### 4.3. Treatment of Spring Wheat Seeds and Germination Test

For seed treatment, spring wheat seeds were dipped into their commercial chitosan and chitosan–silver nanoparticle solutions for 2 h and air-dried under sterile conditions for 24 h before the seed germination test and planting, while deionized water served as a control. Afterwards, the treated seeds were subjected to seed germination tests to assess the influence of Ch-AgNP treatments on the germination parameters of wheat seedlings, including germination percentage, germination energy, root length (RL), and shoot length (SL). A total of 10 treated seeds were placed in each petri dish (5 replicates) and fitted using Whatman No. 1 double filter paper (Merk, Darmstadt, Germany). The germination energy (GE%) was assessed after three days, while germination percentage (GP%), root and shoot lengths (cm) were measured after 14 days. GP% and GE% were calculated according to the following equations:

GP% = (No. of germinated seeds/Total No. of tested seeds) × 100(1)

GE% = (No. of germinated seeds after 3 days/Total No. of tested seeds) × 100(2)

### 4.4. Antifungal Activity Evaluation Against Fusarium graminearum in Spring Wheat

#### 4.4.1. Pot Experiment

Evaluation of the use of different formulations of chitosan–silver nanoparticles (Ch-AgNPs) to control the *Fusarium graminearum* pathogen in spring wheat seeds was conducted using a pot experiment in a controlled growth chamber at the Agrobiology laboratory of the Lithuanian Research Centre for Agriculture and Forestry. Conditions were highly controlled in the growth chamber, with a light intensity of 350 µmole m^−2^ s, a photoperiod of sixteen hours of light and eight hours of night, and a controlled temperature at 24 °C during the day and 16 °C at night, while relative humidity was maintained at 60–70%. Irrigation was applied in accordance with moisture levels to ensure that all treatments received uniform hydration. The pot experiment consisted of a total of ten treatments. Each treatment had five replications and was laid out following a completely randomized design.

Six treatments (1.0 mg/mL) of effective Ch-AgNPs formulations were applied to spring wheat seeds based on preliminary results obtained from in vitro assessment. This concentration showed the highest antifungal activity across all tested formulations, whereas the lower concentrations (0.2, 0.5 mg/mL) showed negligible effects. All treatments of Ch-AgNPs were compared against the following controls: a positive control (soil inoculated with *F. graminearum*, untreated seeds) and a negative control (non-inoculated soil, untreated seeds). Further treatments involved seeds treated with a commercial chitosan solution (1.0 mg/mL) and silver nitrate solution (1.0 mg/mL) for independent evaluation of the influence of chitosan and silver nitrate salt, respectively. Thus, with 5 replicates per treatment and 5 seeds per pot, there were a total of 50 pots: 30 for the Ch-AgNP treatments (6 formulations × 5 replicates) and 5 each for the commercial chitosan, silver nitrate, positive control, and negative control groups. Each pot contained 0.5 kg of sterilized substrate, and the experiment covered the vegetation phase of development in wheat. Thereafter, wheat plants were harvested after 55 days of growth.

#### 4.4.2. Soil Preparation, Fungal Inoculation, and Planting

The growth substrate was prepared by autoclaving compost twice at 121 °C for 15 min to kill native populations of fungi; however, complete sterilization of complex organic materials cannot be guaranteed. At the same time, *Fusarium graminearum* was cultured on potato dextrose agar (PDA) for 14 days at 25 °C under fluorescent light to promote sporulation. The fungal conidial suspension was prepared by gently washing the agar surface with 3 mL of sterile distilled water per Petri plate using an L-shaped cell spreader to dislodge conidia from the mycelium. The suspension was filtered through a 40 µm sterile cell strainer to remove mycelial fragments. Spore concentration was determined using a hemocytometer and adjusted to 1 × 10^6^ spores mL^−1^. A 5 mL aliquot of the suspension was mixed uniformly into each pot designed for pathogen treatment, and then the inoculated soil was incubated for 10 days before sowing. For negative controls, sterile water was added in place of the inoculum. Subsequently, five seeds treated with chitosan–silver nanoparticles were sown per pot (five replicates) in an evenly spaced arrangement.

Observations and data on critical plant health were recorded for assessing the crown and root rot disease severity index (DSI) and incidence of disease based on the disease symptoms of crown rot with browning root scale over the period of 60 days, as described by [47,48]. The *incidence of disease* and *disease severity indices* were calculated based on root and crown discoloration according to the following formulas:

(3) $$Incidence\;of\;disease\;(\%)=\frac{No.of\;infected\;plants}{Total\;No.of\;investigated\;plants}\times 100$$

(4) $$Disease\;severity\;index\;(DSI\%)=⅀100\times \frac{d}{dmax\times n}$$

where d is the sum of disease rating class frequency × score of rating class, dmax is the maximum disease scoring rate, and n is the total number of plants evaluated in each replicate.

### 4.5. Statistical Analysis

All data are expressed as mean ± standard deviation (SDM) of biological replicates. Statistical analyses were performed using IBM SPSS Statistics version 25.0 (IBM Corporation, Armonk, NY, USA). Before analysis, data were evaluated for normal distribution and homogeneity of variances using the Shapiro–Wilk and Levene’s tests, respectively. Comparisons among multiple treatment means were conducted using a one-way analysis of variance (ANOVA), followed by Tukey’s honestly significant difference (HSD) post hoc test at a significance level of *p* < 0.05. Statistical groupings presented as superscript letters in tables and figures were assigned according to Tukey HSD homogeneous subset outputs. All analyses were performed using biological replicates independently prepared for each treatment. Raw data used for statistical analyses are provided in the Supplementary Materials.

## 5. Conclusions and Future Research Directions

This research evaluated the antibacterial and antifungal activity of various formulations of Ch-AgNPs in vitro. Moreover, it assessed their effect on the spring wheat germination parameters and antifungal activity against *F. graminearum* in controlled growth chamber conditions. Among all assessed Ch-AgNPs formulations, L10 and DP4 at 1.0 mg/mL constantly exhibited the highest efficacy in vitro antimicrobial assays, plant growth assays, and in vivo inhibition of *Fusarium graminearum*, which induced fungal and bacterial damage. Hence, it appears that the extraction method, design of chitosan-based nanoparticle formulations, and controlled release of silver ions were crucial in achieving such elevated antimicrobial efficacy combined with reduced phytotoxicity. The findings highlight the potential of optimized Ch-AgNPs formulations as promising alternatives to conventional bactericides and fungicides in integrated pest management systems, especially for soil-borne pathogens in wheat, pending further evaluation of environmental safety and field performance.

Future research work will focus on small-scale field trials to evaluate the range of Ch-AgNP concentrations in order to optimize application rates, assess long-term efficacy, and ensure environmental safety, as well as the efficacy and stability of these formulations under specific agricultural conditions. Particular attention will be paid to their effectiveness in disease suppression, crop yield, and physiological plant responses under various soil and environmental conditions, including different soil types, pH levels, organic matter content, and climatic factors, while also evaluating the stability of the chitosan-based silver nanoparticles with respect to their potential impact on soil microbiota. Moreover, the accumulation of Ch-AgNPs in plant tissues should be investigated, with special emphasis on edible parts such as grains. Furthermore, optimizing the method of application, dosage, and timing will be necessary to achieve efficacy and environmental safety. Such studies will be essential in translating laboratory and controlled chamber experiments to practical solutions for sustainable agriculture.

## Figures and Tables

**Table 1:** Zone of inhibition (ZOI, mm) of commercial chitosan and chitosan–silver nanoparticle (Ch-AgNP) formulations against Gram-negative bacterial strains at 0.2, 0.5, and 1.0 mg/mL.

	*Escherichia coli*			*Xanthomonas campestris*			*Pseudomonas syringae*		
Conc. (mg/mL)	0.2	0.5	1.0	0.2	0.5	1.0	0.2	0.5	1.0
Formulation									
CC	6.25 ± 0.40 ^b^	9.60 ± 0.90 ^a^	10.60 ± 0.50 ^c^	6.0 ± 0.00 ^a^	6.75 ± 0.50 ^c^	11.75 ± 1.92 ^b^	6.17 ± 0.30 ^a^	8.0 ± 0.40 ^c^	14.75 ± 1.50 ^b^
LH1	6.00 ± 0.00 ^b^	8.80 ± 1.40 ^a^	12.30 ± 1.30 ^ab^	6.25 ± 0.50 ^a^	6.75 ± 0.95 ^c^	11.37 ± 1.03 ^b^	6.25 ± 0.50 ^a^	11.75 ± 1.20 ^ab^	17.63 ± 1.10 ^ab^
L10	6.00 ± 0.00 ^b^	9.38 ± 1.30 ^a^	13.63 ± 0.75 ^a^	6.25 ± 0.50 ^a^	12.25 ± 1.55 ^a^	19.37 ± 0.75 ^a^	6.25 ± 0.50 ^a^	12.30 ± 1.60 ^ab^	19.40 ± 0.75 ^a^
L20	8.60 ± 0.30 ^a^	10.00 ± 0.80 ^a^	13.40 ± 0.50 ^a^	6.75 ± 0.50 ^a^	9.00 ± 0.80 ^b^	14.10 ± 0.85 ^b^	7.4 ± 1.80 ^a^	9.90 ± 4.40 ^bc^	18.25 ± 4.60 ^ab^
DP4	6.00 ± 0.00 ^b^	9.25 ± 1.30 ^a^	12.60 ± 1.03 ^ab^	6 ± 0.00 ^a^	8.75 ± 0.95 ^bc^	12.25 ± 0.50 ^b^	7.87 ± 1.10 ^a^	15.25 ± 2.60 ^a^	19.90 ± 1.50 ^a^
AS3	6.00 ± 0.00 ^b^	8.60 ± 0.48 ^a^	10.80 ± 0.55 ^bc^	6 ± 0.00 ^a^	8.00 ± 0.00 ^bc^	11.75 ± 3.00 ^b^	7.50 ± 0.00 ^a^	14.63 ± 1.00 ^a^	20.90 ± 0.50 ^a^

Values are expressed as mean ± standard deviation of four replicates (*n* = 4). Different superscript letters within the same column indicate significant differences among treatments as determined by one-way ANOVA followed by Tukey’s HSD test (*p* < 0.05).

**Table 2:** Zone of inhibition (ZOI, mm) of commercial chitosan and chitosan–silver nanoparticle (Ch-AgNP) formulations against *Staphylococcus aureus* at 0.2, 0.5, and 1.0 mg/mL.

*Staphylococcus aureus*			
Conc. (mg/mL)	0.2	0.5	1.0
Formulation			
CC	6.00 ± 0.00 ^a^	6.00 ± 0.0 ^c^	7.50 ± 0.50 ^b^
LH1	6.00 ± 0.00 ^a^	6.50 ± 0.58 ^c^	8.75 ± 1.90 ^b^
L10	6.00 ± 0.00 ^a^	6.00 ± 0.00 ^c^	12.62 ± 1.25 ^a^
L20	6.00 ± 0.00 ^a^	6.5 ± 0.57 ^c^	10.00 ± 2.80 ^ab^
DP4	6.00 ± 0.00 ^a^	9.00 ± 0.00 ^a^	13.00 ± 0.81 ^a^
AS3	6.50 ± 0.57 ^a^	7.80 ± 0.55 ^b^	8.75 ± 0.64 ^b^

Values are expressed as mean ± standard deviation of four replicates (*n* = 4). Different superscript letters within the same column indicate significant differences among treatments as determined by a one-way ANOVA followed by Tukey’s HSD test (*p* < 0.05).

**Table 3:** Mycelium diameter and growth inhibition (%) of chitosan–silver nanoparticle formulations against *F. graminearum* at 0.2, 0.5, 1.0 mg/mL.

Mycelium Diameter				Growth Inhibition (%)		
Conc. (mg/mL)	0.2	0.5	1.0	0.2	0.5	1.0
Formulation						
CC	80.00 ± 0.00 ^a^	66.00 ± 18.49 ^b^	32± 7.36 ^b^	0.00 ^a^	17.50 ^ab^	60.00 ^b^
LH1	80.00 ± 0.00 ^a^	80.00 ± 0.00 ^b^	74.75 ± 4.27 ^b^	0.00 ^a^	0.00 ^c^	7.00 ^c^
L10	80.00 ± 0.00 ^a^	48.00 ± 14.09 ^a^	6.50 ± 13.00 ^a^	0.00 ^a^	40.00 ^a^	92.00 ^a^
L20	80.00 ± 0.00 ^a^	80.00 ± 0.00 ^b^	14.75 ± 18.10 ^ab^	0.00 ^a^	0.00 ^c^	81.56 ^ab^
DP4	80.00 ± 0.00 ^a^	73.00 ± 6.16 ^b^	12.50 ± 17.45 ^ab^	0.00 ^a^	8.75 ^b^	84.00 ^ab^
AS3	80.00 ± 0.00 ^a^	80.00 ± 0.00 ^b^	38.00 ± 43.95 ^c^	0.00 ^a^	0.00 ^c^	52.50 ^b^

Values are expressed as mean ± standard deviation of four replicates (*n* = 4). Different superscript letters within the same column indicate significant differences among treatments as determined by one-way ANOVA followed by Tukey’s HSD test (*p* < 0.05).

**Table 4:** Categorization of chitosan–silver nanoparticle formulations.

Sample Abbreviation	Definition
CC	Commercial chitosan
CC_NP_	Chitosan–silver nanoparticles obtained from commercial chitosan (CC)
LH1	Chitosan–silver nanoparticles were obtained from chitosan extracted via 1% lactic acid, 4% NaOH, and 50% NaOH at room temperature (RT) for demineralization, deproteinization, and deacetylation, respectively.
L10	Chitosan–silver nanoparticles were obtained from chitosan extracted via 10% lactic acid, 4% NaOH, and 50% NaOH at 50 °C for demineralization, deproteinization, and deacetylation, respectively.
L20	Chitosan–silver nanoparticles were obtained from chitosan extracted via 20% lactic acid, 4% NaOH, and 50% NaOH at 50 °C for demineralization, deproteinization, and deacetylation, respectively.
AS3	Chitosan–silver nanoparticles were obtained from chitosan extracted via 3% acetic acid, 4% NaOH, and 50% NaOH at 50 °C for demineralization, deproteinization, and deacetylation, respectively.
DP4	Chitosan–silver nanoparticles were obtained from chitosan extracted via 4% NaOH, 2% HCl, and 50% NaOH at 50 °C for deproteinization, demineralization, and deacetylation, respectively.

Each stage was conducted under controlled conditions of a 1:10 (*w*/*v*) solid-to-liquid ratio, 150 rpm stirring for 2 h, followed by an additional soaking period of 24 h without agitation for the deproteinization and deacetylation stages, respectively.

## Data Availability

Data available upon request.

## Supplementary Materials

The following supporting information can be downloaded at: https://www.mdpi.com/article/10.3390/md24050183/s1, Table S1: Zone of inhibition (ZOI, mm) of commercial chitosan and chitosan–silver nanoparticle (Ch-AgNP) formulations against Gram-negative bacterial strains at 0.2, 0.5, and 1.0 mg/mL; Table S2: Zone of inhibition (ZOI, mm) of commercial chitosan and chitosan–silver nanoparticle (Ch-AgNP) formulations against Staphylococcus aureus at 0.2, 0.5, and 1.0 mg/mL; Table S3: Mycelium diameter and growth inhibition (%) of chitosan–silver nanoparticle formulations against *F. graminearum* at 0.2, 0.5, 1.0 mg/mL; Table S4: For Figure 2, Germination percentage of wheat seedlings at 1.0 mg/mL; Table S5: For Figure 3, Shoot and root lengths (cm) of wheat seedlings at 1.0 mg/mL; Table S6: For Figure 4, Incidence and disease severity indices of *Fusarium graminearum* in spring wheat under Ch-AgNP formulations at 1.0 mg/mL.
